# Supplementary material for: Characterization of cephalic and non-cephalic sensory cell types provides insight into joint photo- and mechanoreceptor evolution
Source: eLife. 2021 Aug 5;10:e66144. doi: 10.7554/eLife.66144 (PMC8367381; doi:10.7554/eLife.66144)
Supplement: Figure 3—source data 1. — Best P. dumerilii BLAST hits of the corresponding D. melanogaster genes (middle column), as described in detail in Materials and methods. The yellow shading indicates genes that are part of the D. melanogaster phototransduction pathway. [file elife-66144-fig3-data1.pdf]

Group	*P. dumerilii* Gene ID	*D. melanogaster* Gene Symbol	BLAST E value
EP-SPECIFIC	c2720	*gαi*	1e-165
EP-SPECIFIC	c680	*dnc*	0
EP-SPECIFIC	c8760	*cg3501*	2e-40
EP-SPECIFIC	c10874	*numb*	5e-117
EP-SPECIFIC	c9898	*ipp*	2e-75
EP-SPECIFIC	c8120	*inac*	0
COMMON EP / TRE	c10788	*trp/trpl*	6e-129/1e-142
COMMON EP / TRE	c10763	*gβ76c*	2e-101
COMMON EP / TRE	c3283	*rh2*	6e-62
COMMON EP / TRE	c10800	*norpa*	1e-129
COMMON EP / TRE	c6424	*gαq/cg30054/cta*	0/7e-156/2e-78
COMMON EP / TRE	c10413	*hook*	4e-107
COMMON EP / TRE	c10969	*cg5027*	3e-48
COMMON EP / TRE	c8541	*psn*	1e-104
COMMON EP / TRE	c10958	*rapgap1*	4e-157
TRE-SPECIFIC	c4259	*ds/cad99C*	1e-91/1e-52
TRE-SPECIFIC	c9464	*cg3887*	2e-44
TRE-SPECIFIC	c10745	*cg5168*	1e-164
TRE-SPECIFIC	c2053	*gl*	1e-70
TRE-SPECIFIC	c11125	*cg11880*	8e-49
TRE-SPECIFIC	c14153	*cg7149*	2e-99
TRE-SPECIFIC	c19480	*cg3356*	5e-46
TRE-SPECIFIC	c7820	*ap1sigma*	3e-98

Figure 3- source data 1
