# Supplementary material for: Characterization of cephalic and non-cephalic sensory cell types provides insight into joint photo- and mechanoreceptor evolution
Source: eLife. 2021 Aug 5;10:e66144. doi: 10.7554/eLife.66144 (PMC8367381; doi:10.7554/eLife.66144)
Supplement: Figure 3—source data 2. — Each gene in the ‘P. dumerilii Gene ID’ column indicates the best P. dumerilii BLAST hit to the corresponding M. musculus. Gene symbols in the middle column, as described in detail in Materials and methods. The yellow shading indicates genes that are involved in the sensory perception of sound (Figure 3J). [file elife-66144-fig3-data2.pdf]

Group	*P. dumerilii* Gene ID	*D. melanogaster* Gene Symbol	BLAST E value
EP-SPECIFIC	c19864	Ano1	4e-22
EP-SPECIFIC	c6662	Grin2a/b/d	2e-42/4e-44/3e-4[illegible]
EP-SPECIFIC	c11624	Cdh23/Pcdh15	2e-157/2e-52
EP-SPECIFIC	c1216	Chrna10	2e-56
EP-SPECIFIC	c15086	Lrig1	1e-21
EP-SPECIFIC	c29499	Tub	6e-152
EP-SPECIFIC	c36304	Slc12a2	4e-41
EP-SPECIFIC	c7424	Atp2b2	0
EP-SPECIFIC	c10941	Gabrb2/3	2e-74/1e-75
EP-SPECIFIC	C7778	Atp6v1b1	5e-108
COMMON EP / TRE	c10559	Rab3a	1e-121
COMMON EP / TRE	c19136	Sod2	1e-85
COMMON EP / TRE	c10364	Wdr1	7e-134
COMMON EP / TRE	c10709	Tbl1x	0
COMMON EP / TRE	c10848	Clic5	6e-30
COMMON EP / TRE	c10850	Sptbn4	3e-98
COMMON EP / TRE	c10955	Kcnma1	0
COMMON EP / TRE	c1540	Map1a	8e-83
COMMON EP / TRE	c30248	Rpl38	9e-24
COMMON EP / TRE	c3250	Atp6v0a4	0
COMMON EP / TRE	c34334	Homer2	1e-91
COMMON EP / TRE	C4259	Cdh1	1e-21
COMMON EP / TRE	c6138	Fgfr1/Fyn/Kit	6e-54/0/1e-34
COMMON EP / TRE	c6400	Casp3	5e-70
COMMON EP / TRE	c9994	Sod1	7e-63
COMMON EP / TRE	c8074	Myo7a/15/1a	2e-148/3e-136/0
COMMON EP / TRE	c13777	Myo6	0
TRE-SPECIFIC	c11426	Ntrk1	2e-55
TRE-SPECIFIC	c11895	Chrna9	1e-92
TRE-SPECIFIC	c23606	Crym	4e-109
TRE-SPECIFIC	c2513	Whrn	2e-22
TRE-SPECIFIC	c28439	Serpinb6a	2e-70
TRE-SPECIFIC	c36224	Axin1	9e-36
TRE-SPECIFIC	c4523	Sox2	5e-33
TRE-SPECIFIC	c4683	Mkks	3e-60
TRE-SPECIFIC	c5186	Dnm1	0
TRE-SPECIFIC	c6103	Myh14	0
TRE-SPECIFIC	c10567	Atp8b1	1e-97
TRE-SPECIFIC	c10606	Jag2	5e-70
TRE-SPECIFIC	c11252	Scn8a	3e-137
TRE-SPECIFIC	c13113	Snai2	1e-24
TRE-SPECIFIC	c14655	Myo3a	0
TRE-SPECIFIC	c20437	Tecta	1e-38
TRE-SPECIFIC	c35565	Hoxa1	1e-20
TRE-SPECIFIC	c3629	Ush2a	2e-34
TRE-SPECIFIC	c7677	Trpa1/Espn	8e-94/2e-26

Figure 3- source data 2
