# Supplementary material for: Characterization of cephalic and non-cephalic sensory cell types provides insight into joint photo- and mechanoreceptor evolution
Source: eLife. 2021 Aug 5;10:e66144. doi: 10.7554/eLife.66144 (PMC8367381; doi:10.7554/eLife.66144)
Supplement: Supplementary file 1. [file elife-66144-supp1.docx]

| *Gene/ID* | Enrichment in EP cells (FDR) | Enrichment in TRE cells (FDR) | Category | Data/validation |
| --- | --- | --- | --- | --- |
| *r-opsin1/c8630* | **5x10^-3^** | **8x10^-4^** | common EP/TRE | Fig. 1E, ref. (Backfisch et al., 2013) |
| *egfp/c13611* | **7x10^-3^** | **4x10^-3^** | common EP/TRE | Fig. 1F, ref. (Backfisch et al., 2013) |
| *gq/c6424* | **0.018** | **0.010** | common EP/TRE | Fig. 1G, ref. (Backfisch et al., 2013) |
| *ngbl/c10609* | **7x10^-3^** | **7x10^-3^** | common EP/TRE | Fig. 2 – figure supplement 1A, 3E-H, 4B-C |
| *tmdc/c2433* | **7x10^-3^** | **7x10^-3^** | common EP/TRE | Fig. 2 – figure supplement 1B, ref. (Pende et al., 2020) |
| *f8a/c6996* | -0.12 | **0.019** | TRE-specific | Fig. 2 – figure supplement 1C, 3K-L, 4H-I |
| *dmd/c7924* | 0.70 | **0.019** | TRE-specific | Fig. 2 – figure supplement 1D, 3M-N, 4J-K |
| *trpA/c7677* | -0.45 | **0.038** | TRE-specific | Fig. 2 – figure supplement 1E, 3I-J, 4F-G, Fig. 3E |
| *rps9/c34148* | -0.99 | -0.64 | not enriched | Fig. 1 – figure supplement 1C |
| *cdc5l/c20710* | 0.61 | -0.39 | not enriched | Fig. 1 – figure supplement 1D |

**Fig.2- figure supplement 2. Synopsis of validated genes identified in the transcriptome profiling.**
