# Supplementary material for: Characterization of cephalic and non-cephalic sensory cell types provides insight into joint photo- and mechanoreceptor evolution
Source: eLife. 2021 Aug 5;10:e66144. doi: 10.7554/eLife.66144 (PMC8367381; doi:10.7554/eLife.66144)
Supplement: Supplementary file 2. [file elife-66144-supp2.docx]

| Gene name | Gene ID | Genbank Accession | Gene symbol of homolog mentioned in this manuscript |
| --- | --- | --- | --- |
| *r-opsin1* | *c8630* | AJ316544.1 | NinaE/Rh3/Rh4 (*D. melanogaster*) |
| *egfp* | *c13611* | LC336974.1 |  |
| *gq* | *c6424* | KC109635.1 | Galphaq (*D. melanogaster*) |
| *ngbl* | *c10609* | MT444158 [this study] |  |
| *tmdc* | *c2433* | MK330892 |  |
| *f8a* | *c6996* | MT444159 [this study] |  |
| *dmdl* | *c7924* | MT444160 [this study] |  |
| *trpA* | *c7677* | MT444161 [this study] |  |
| *rps9* | *c34148* | KF606862.1 |  |
| *cdc5l* | *c20710* | GU322430.1 |  |
| *r-opsin3* | *c3283* | KC810971.1 | Rh5/Rh6 (*D. melanogaster*) |
| *gnb* | *c10763* | MT444162 [this study] | Gbeta76C (*D. melanogaster*) |
| *gngl* | *c33855* | MT444163 [this study] | Ggamma30A (*D. melanogaster*) |
| *plcb* | *c10800* | MT444164 [this study] | NorpA (*D. melanogaster*) |
| *prkc* | *c8120* | MT444165 [this study] | InaC (*D. melanogaster*) |
| *trpc* | *c10788* | MT444166 [this study] | Trp/Trpl (*D. melanogaster*) |
| *mpdzl* | *c7982* | MT444167 [this study] | InaD (*D. melanogaster*) |
| *calml* | *c7432* | MT444168 [this study] | Cam (*D. melanogaster*) |
| *myo3* | *c14655* | MT444169 [this study] | NinaC (*D. melanogaster*) |
| *tnikl* | *c8565* | MT444170 [this study] |  |
| *arrb* | *c8733* | MT444171 [this study] | Arr2 (*D. melanogaster*) |
| *pip5k1* | *c10883* | MT444172 [this study] | PIP5K59B (*D. melanogaster*) |
| *whrn* | *c2513* | MT444173 [this study] | Whrn (*M. musculus*) |
| *dnm* | *c5186* | MT444174 [this study] | Dnm1 (*M. musculus*) |
| *atp9l* | *c10567* | MT444175 [this study] | Atp8b1 (*M. musculus*) |
| *chrna9* | *c11895* | MT444176 [this study] | Chrna9 (*M. musculus*) |
| *tecta* | *c20437* | MT444177 [this study] | Tecta (*M. musculus*) |
| *soxc* | *c4523* | FN357282.1 | Sox2 (*M. musculus*) |
| *notch* | *c10606* | MT444178 [this study] | Jag2 (*M. musculus*) |
| *crym* | *c23606* | MT444179 [this study] | Crym (*M. musculus*) |
| *serpinbl* | *c28439* | MT444180 [this study] | Serpinb6a (*M. musculus*) |
| *myh10l* | *c6103* | MT444181 [this study] | Myh14 (*M. musculus*) |
| *atp2b* | *c7424* | MT444182 [this study] | Atp2b2 (*M. musculus*) |
| *pkd2.1* | *NA* | MZ647696 [this study] | See Bezares-Calderón *et. al.* (2018) |
| *nompc* | *NA* | MZ647694 [this study] | NompC (*D. melanogaster*) |
| *piezo* | *NA* | MZ647695 [this study] | Piezo (*D. melanogaster*) |
| *tmc123* | *NA* | MZ747700 [this study] | Tmc1/2/3 (*M. musculus*) |

**Fig.2- figure supplement 5. Sequence identifiers of *Platynereis* genes analyzed in this study.**
