# Supplementary material for: Characterization of cephalic and non-cephalic sensory cell types provides insight into joint photo- and mechanoreceptor evolution
Source: eLife. 2021 Aug 5;10:e66144. doi: 10.7554/eLife.66144 (PMC8367381; doi:10.7554/eLife.66144)
Supplement: Transparent reporting form [file elife-66144-transrepform.docx]

***eLife’s* transparent reporting form**

We encourage authors to provide detailed information *within their submission* to facilitate the interpretation and replication of experiments. Authors can upload supporting documentation to indicate the use of appropriate reporting guidelines for health-related research (see [EQUATOR Network](http://www.equator-network.org/%20)), life science research (see the [BioSharing Information Resource](https://biosharing.org/)), or the [ARRIVE guidelines](http://www.plosbiology.org/article/info:doi/10.1371/journal.pbio.1000412) for reporting work involving animal research. Where applicable, authors should refer to any relevant reporting standards documents in this form.

If you have any questions, please consult our Journal Policies and/or contact us: [editorial@elifesciences.org](mailto:editorial@elifesciences.org).

**Sample-size estimation**

- You should state whether an appropriate sample size was computed when the study was being designed
- You should state the statistical method of sample size computation and any required assumptions
- If no explicit power analysis was used, you should describe how you decided what sample (replicate) size (number) to use

Please outline where this information can be found within the submission (e.g., sections or figure legends), or explain why this information doesn’t apply to your submission:

No explicit power analysis was used. Sample sizes were selected for each experiment type as detailed below:

Transcriptome profiling: Three biological replicates were carried out. This is according to the standards of the field (and due to time and material constraints). For details see Materials and Methods section and Fig.1E-G.

Differential expression analysis: Three biological replicates were carried out. This is according to the standards of the field (and due to time and material constraints). For details see Materials and Methods section and Fig.2B.

G-protein coupling assay and action spectrum: Each test was carried out in three independent experiments, according to the standards of the field and published work. For details see Materials and Methods and Fig.5A, Fig.5- figure supplement 1.

Behavioral analyses: Sample size was based on previous observations and publications regarding the expected variability of individual worms. Trial-to-trial variability could of course be reduced by averaging across more individuals.

For details see Materials and Methods and Fig.6F-H, Fig.6- figure supplement 2.

**Replicates**

- You should report how often each experiment was performed
- You should include a definition of biological versus technical replication
- The data obtained should be provided and sufficient information should be provided to indicate the number of independent biological and/or technical replicates
- If you encountered any outliers, you should describe how these were handled
- Criteria for exclusion/inclusion of data should be clearly stated
- High-throughput sequence data should be uploaded before submission, with a private link for reviewers provided (these are available from both GEO and ArrayExpress)

Please outline where this information can be found within the submission (e.g., sections or figure legends), or explain why this information doesn’t apply to your submission:

Transcriptome profiling: Three biological replicates were carried out. This is according to the standards of the field (and due to time and material constraints). For details see Materials and Methods section and Fig.1E-G.

Biological replicates: independent experiments with different animals on different dates

Differential expression analysis: Three biological replicates were carried out. This is according to the standards of the field (and due to time and material constraints). For details see Materials and Methods section and Fig.2B.

Biological replicates: independent experiments with different animals on different dates

G-protein coupling assay and action spectrum: Each test was carried out in three independent experiments, according to the standards of the field and published work. For details see Materials and Methods and Fig.5A, Fig.5- figure supplement 1.

Biological replicates: independent experiments with different wells of cells.

Behavioral analyses:

– intense light avoidance assay: For details see Materials and Methods and Fig.6- figure supplement 2.

Biological replicates: individual decapitated worms (each animal was only used in one experiment)

– undulation assay: For details see Materials and Methods and Fig.6E-H.

Biological replicates: individual decapitated worms (each animal was only used in one experiment), in total the experiments were performed at three independent times, spaced apart by several weeks.

Outliers: worms that showed signs of maturation any time during the experiment or showed convulsions or that could not be properly tracked were excluded from the analyses. We also excluded two batches of experiments where we realized that our sea water had gone bad and killed larvae in the culture.

All data and code can be downloaded from DRYAD: <https://datadryad.org/stash/share/_AWaPRHfmuHKyq9CzHt7YNtmaAgHFQXjfklkVE1n6SI>.

doi:10.5061/dryad.m63xsj416

**Statistical reporting**

- Statistical analysis methods should be described and justified
- Raw data should be presented in figures whenever informative to do so (typically when N per group is less than 10)
- For each experiment, you should identify the statistical tests used, exact values of N, definitions of center, methods of multiple test correction, and dispersion and precision measures (e.g., mean, median, SD, SEM, confidence intervals; and, for the major substantive results, a measure of effect size (e.g., Pearson's r, Cohen's d)
- Report exact p-values wherever possible alongside the summary statistics and 95% confidence intervals. These should be reported for all key questions and not only when the p-value is less than 0.05.

Please outline where this information can be found within the submission (e.g., sections or figure legends), or explain why this information doesn’t apply to your submission:

Differential expression analysis: see Methods- Differentially expressed genes.

Gene set overlap analyses: see Methods- Statistical assessment of subset specificity.

Enrichment of atp2b2 mRNA expression in zebrafish neuromasts cells: Student’s t-test with equal variance on the QPCR cycle number difference (according to Bartlett’s test), as cycle number difference shows normal distribution. For details see Fig. 6 – figure supplement 1 and Methods (section “Enrichment of atp2b2 mRNA expression in zebrafish neuromasts cells”).

Behavior: see Methods- Behavior

- strong light avoidance: Wilcoxon rank sum test (general test that is reliable, irrespective of data distribution)

- undulation analysis: First, from the undulation ratios the area under the curve was calculated for every replicate and then the datasets were tested for normal distribution (Shapiro-Wilk normality test). To determine if there were differences between the groups, either a paired (light versus dark) Wilcoxon signed rank test or an unpaired (wildtype versus mutant) Wilcoxon rank sum test was conducted.

As not all samples/differences of paired samples were normally distributed, the Wilcoxon tests were used as alternative to Student's t-tests. Light vs. dark samples within the genotype are related samples as the experiment was continuous and thus the same set of animals was measured.

(For large datasets, or papers with a very large number of statistical tests, you may upload a single table file with tests, Ns, etc., with reference to sections in the manuscript.)

**Group allocation**

- Indicate how samples were allocated into experimental groups (in the case of clinical studies, please specify allocation to treatment method); if randomization was used, please also state if restricted randomization was applied
- Indicate if masking was used during group allocation, data collection and/or data analysis

Please outline where this information can be found within the submission (e.g., sections or figure legends), or explain why this information doesn’t apply to your submission:

Animals were randomly chosen from the culture and -where applicable- grouped according to genotype.

**Additional data files (“source data”)**

- We encourage you to upload relevant additional data files, such as numerical data that are represented as a graph in a figure, or as a summary table
- Where provided, these should be in the most useful format, and they can be uploaded as “Source data” files linked to a main figure or table
- Include model definition files including the full list of parameters used
- Include code used for data analysis (e.g., R, MatLab)
- Avoid stating that data files are “available upon request”

Please indicate the figures or tables for which source data files have been provided:

Figures 1-3,5,6, Fig. 1- figure supplement 1, Fig. 2- figure supplement 1, Fig. 2- supplementary files 1,2, Fig.3- source data 1,2, Fig.5- figure supplement 1, Fig.6- figure supplement 3

All data and code can be downloaded from DRYAD: <https://datadryad.org/stash/share/_AWaPRHfmuHKyq9CzHt7YNtmaAgHFQXjfklkVE1n6SI>.

doi:10.5061/dryad.m63xsj416
